# Promotion of Ca^2+^ Accumulation in Roots by Exogenous Brassinosteroids as a Key Mechanism for Their Enhancement of Plant Salt Tolerance: A Meta-Analysis and Systematic Review

**DOI:** 10.3390/ijms242216123

**Published:** 2023-11-09

**Authors:** Xian Wang, Jiali Chai, Wenyu Liu, Xiaolin Zhu, Haixun Liu, Xiaohong Wei

**Affiliations:** 1 Agronomy College, Gansu Agricultural University, Lanzhou 730070, China; wx1431268954@163.com (X.W.); zxl81724@163.com (X.Z.); 2 Gansu Provincial Key Laboratory of Aridland Crop Science, Lanzhou 730070, China; 3 Gansu Key Laboratory of Crop Genetic & Germplasm Enhancement, Lanzhou 730070, China; 4 Pratacultural College, Gansu Agricultural University, Lanzhou 730070, China; 5 Gansu Academy of Agricultural Sciences, Lanzhou 730070, China; 6 College of Life Science and Technology, Gansu Agricultural University, Lanzhou 730070, China

**Keywords:** salt stress, brassinosteroids, meta-analysis, physiological

## Abstract

Brassinosteroids (BRs), the sixth major phytohormone, can regulate plant salt tolerance. Many studies have been conducted to investigate the effects of BRs on plant salt tolerance, generating a large amount of research data. However, a meta-analysis on regulating plant salt tolerance by BRs has not been reported. Therefore, this study conducted a meta-analysis of 132 studies to elucidate the most critical physiological mechanisms by which BRs regulate salt tolerance in plants from a higher dimension and analyze the best ways to apply BRs. The results showed that exogenous BRs significantly increased germination, plant height, root length, and biomass (total dry weight was the largest) of plants under salt stress. There was no significant difference between seed soaking and foliar spraying. However, the medium method (germination stage) and stem application (seedling stage) may be more effective in improving plant salt tolerance. BRs only inhibit germination in Solanaceae. BRs (2 μM), seed soaking for 12 h, and simultaneous treatment with salt stress had the highest germination rate. At the seedling stage, the activity of Brassinolide (C_28_H_48_O_6_) was higher than that of Homobrassinolide (C_29_H_50_O_6_), and post-treatment, BRs (0.02 μM) was the best solution. BRs are unsuitable for use in the germination stage when Sodium chloride is below 100 mM, and the effect is also weakest in the seedling stage. Exogenous BRs promoted photosynthesis, and antioxidant enzyme activity increased the accumulation of osmoregulatory and antioxidant substances and reduced the content of harmful substances and Na^+^, thus reducing cell damage and improving plant salt tolerance. BRs induced the most soluble protein, chlorophyll a, stomatal conductance, net photosynthetic rate, Glutathione peroxidase, and root-Ca^2+^, with BRs causing Ca^2+^ signals in roots probably constituting the most important reason for improving salt tolerance. BRs first promoted the accumulation of Ca^2+^ in roots, which increased the content of the above vital substances and enzyme activities through the Ca^2+^ signaling pathway, improving plant salt tolerance.

## 1. Introduction

Salt damage is one of the main factors affecting plant growth, yield, and quality. In recent years, saline land has been expanding due to climate change, wrong irrigation, indiscriminate cutting, the unreasonable use of chemical fertilizers, and numerous natural factors, including climate, water quality, and terrain reasons [1,2,3]. To date, the saline land area in the world has reached 800 million hectares, continues to increase, and has exceeded 5% of the total land area. The saline problem is endangering the development of modern agriculture [4]. The adverse effects of salt stress on plants are multifaceted, including water deficiency due to osmotic stress, plant cell ion homeostasis disruption, ion toxicity from Na^+^ and Cl^−^, and acute K^+^ deficiency due to K^+^ leakage [5]. In addition, the accumulation of reactive oxygen species (ROS) in plants, photosynthesis, and metabolism are also inhibited under salt stress [6,7]. This series of adverse factors eventually leads to stunted growth, reduced biomass and yield, accelerated senescence, and even the death of plants [8,9].

Brassinosteroids (BRs) are a class of polyhydroxylated sterols, the sixth major phytohormone critical in plant growth and development [10]. Grove et al. first isolated and characterized the molecular structure of this hormone [11]. Subsequent researchers successfully synthesized and isolated a variety of BRs from nature and found that they could regulate rootstock growth, seed development, photosynthesis, protein and nucleic acid synthesis, and various enzyme activities [3]. In recent years, there has been increasing evidence that BRs also play positive roles to varying degrees in abiotic adversities such as plant water stress [12], cold stress [13], heavy metal stress [14], and especially salt stress [15,16]. Thus, BRs are often used to enhance salt tolerance in plants. the exogenous application of BRs can improve salt tolerance by increasing the antioxidant enzyme activity, photosynthesis, and osmoregulatory capacity, improving ion homeostasis, and reducing membrane lipid peroxidation in cells, thus promoting better plant growth and development under salt stress [3]. For example, He et al. found that the exogenous 24-epibrassinolide (EBR) could modulate photosynthetic pigments, antioxidant defense systems, ion homeostasis, osmoregulatory substances, and activate salt tolerance-related signaling pathways to improve salt tolerance in cucumber [17]. To date, many researchers have successfully used BRs to increase salt tolerance in plants. It was found that salt stress specifically triggers primary calcium signaling in Arabidopsis root differentiation zones, which enhances Na^+^ exclusion and plant salt tolerance [18]. In addition, Ca^2+^ application also increased the superoxide dismutase activity, catalase activity, chlorophyll content, and root activity in cereal grains, thereby reducing the injury caused by salt stress [19]. In conclusion, Ca^2+^ plays an important role in plant salt tolerance.

Although many studies have been published on the enhancement of plant salt tolerance by BRs, these studies are often stand-alone and thus cannot elucidate the exact mechanism by which BRs enhance plant salt tolerance in a higher dimension. The usage of BRs and the mechanisms by which BRs improve salt tolerance remain variable and debated. Both Chen et al. and Sharma et al. exogenously applied BRs to rice [20,21]. However, they reached opposite conclusions regarding the effect of BRs on CAT activity. In addition to this, the effects of exogenous BRs on ascorbate peroxidase (APX), peroxidase (POD), intercellular CO_2_ concentration (Ci), transpiration rate (E), K^+^, and maximal photochemical efficiency (Fv/Fm) in plants under salt stress have been inconsistent in different studies. To date, the most appropriate application method of BRs in increasing salt stress tolerance in plants and the differences in the response of different classifications of plants to BRs under salt stress are unknown. What role does Ca^2+^ play in regulating plant salt tolerance by BRs? Meta-analysis is a systematic and comprehensive analysis process that combines data from the multiple experiments of the same type to reach an accurate and comprehensive conclusion, essentially solving the aforementioned problems [22,23]. Wang et al. [24] investigated the effects of melatonin on drought stress in plants by meta-analysis, which showed that the positive effects of melatonin on biomass and chlorophyll were diminished when the concentration range of melatonin was higher than 80–120 µmol L^−1^ and that the effects of soil application were more pronounced than those of foliar spraying. Tahjib et al. [25] similarly used meta-analysis to conclude that the optimal concentration range for nitric oxide to alleviate salt stress (150 mM NaCl) in plants is 0.1–0.2 mM. Exogenous nitric oxide improves plant salt tolerance by alleviating oxidative damage, promoting photosynthesis, and improving ion homeostasis. However, the meta-analysis of the regulation of salt tolerance in plants by BRs has yet to be reported. For this reason, we screened 132 relevant studies for meta-analysis to evaluate the effects of exogenous BRs on plant growth, antioxidant substances, photosynthesis, ionic changes, and other standard physiological parameters under salt stress. This study will provide theoretical guidance and data support for applying BRs under salt stress and provide a more accurate understanding of the physiological mechanisms by which BRS regulates salt tolerance in plants. This study aimed to answer the following questions:

1. What is the best application strategy for BRs to improve seed germination rate?
2. What are the best donor compounds (C_29_H_50_O_6_ or C_28_H_48_O_6_), concentrations, and application methods for exogenous BRs in the seedling stage?
3. What are the differences between exogenous BRs in alleviating different levels of salt stress?
4. Does the effect of exogenous BRs on plant salt tolerance vary with plant taxonomy (monocotyledon vs. dicotyledon; herbaceous vs. woody)?
5. How do exogenous BRs affect plant growth (plant height, root length, and biomass), antioxidant substances (ascorbic acid (ASA), reduced glutathione (GSH), dehydroascorbate reductase (DHAR), superoxide dismutase (SOD), guaiacol peroxidase (POX), POD, glutathione reductase (GR), glutathione peroxidase (GPX), catalase (CAT), and APX), photosynthesis (chlorophyll content, net photosynthetic rate (Pn), E, stomatal conductance (Gs), and Ci), ion changes (Ca^2+^, Na^+^, K^+^, and Mg^2+^), and other standard physiological parameters? Which physiological processes are most important in BRs increasing the plant salt tolerance levels?

## 2. Materials and Methods

### 2.1. Literature Screening

Since PROSPERO is limited to registering health-related research, this study was registered in the Open Science Framework (OSF) on 26 September 2023, at https://osf.io/zcqy9 (accessed on 26 September 2023), with a digital object identifier (DOI) of 10.17605/OSF.IO/ZCQY9. The OSF is an open source platform to share the review with others transparently and assist researchers throughout the review process. In this study, research papers were collected from the Web of Science, PubMed, CNKI, China Science and Technology Journal Database, and WanFang Database, and the search deadline was 9 February 2023. An advanced search form was used, i.e., subject terms + free words, and the search form for each database is detailed in Supplementary File S1.

**Figure 1:**
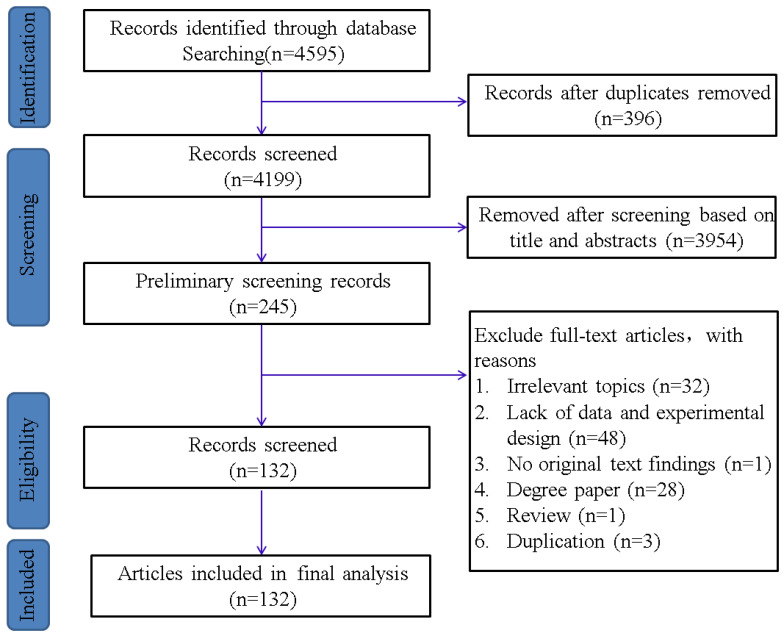
Flow chart of search and exclusion for meta-analysis studies.

To ensure the validity of the collected literature, the following selection criteria were defined: (1) all studies are from journal papers, and experiments must be conducted in non-field conditions. (2) The experimental material must be wild-type, and there must be separate NaCl and NaCl + BRs(Brassinolide (C_28_H_48_O_6_) and Homobrassinolide (C_29_H_50_O_6_)) treatments in the experiment, with the donor of BRs excluding its analogs. (3) The experimental data must include at least one predetermined and identifiable physiological parameter. (4) The experimental results must include complete and valid data, including at least the mean, error values, and the number of replications. (5) Must have clearly described experimental methods. (6) Excluding the reviews and apology letters, duplicate published or cited data will be removed. Based on the above guidelines, 4595 research papers were found, and 132 (4740 independent studies) (Supplementary File S3) were finally obtained for subsequent meta-analysis after step-by-step screening (Figure 1).

### 2.2. Data Extraction and Classification

An Excel database was created to extract valid data from the paper. The extracted data included mean, standard deviation (SD), number of replicates, and experimental description information (Table S1). Data in image form were accurately extracted using the online website WebPlotDigitizer (https://automeris.io/WebPlotDigitizer/index.html (accessed on 25 March 2023)). When standard errors (SEs) are given in this paper, the SD value is obtained using the formula SD = SE × $\sqrt{sample\;size}$. To improve the study’s accuracy, we hypothesized the individual studies of each paper as independent experiments [26]. For analysis, studies were coded categorically, such as plant groups, plant types, family, salt stress level (high, >150 mM; medium, 100–150 mM; low, <100 mM), BR level (high, >1 μM; medium, 0.02–1 μM; low, <0.02 μM), application method, treatment time, and donor (Table S1 attached).

### 2.3. Meta-Analysis

Meta-analysis was performed using MetaWin 2.1 software, lnR was selected as the effect value type, and the effect size and combined effect values were calculated. A random-effects model was used in the presence of heterogeneity (*p* < 0.05), and a fixed-effects model was used in the opposite direction [27,28]. The effect values of each data group were first subjected to the K-S test. When the data did not conform to a normal distribution, the corrected confidence interval bootstrap CI was selected for data analysis, and vice versa; the 95% CI was chosen for data analysis [29]. When the confidence interval crosses the zero line, the difference between the control and treatment groups is considered insignificant, and conversely, the difference is significant. Non-overlapping confidence intervals for the different subgroup analyses were considered significant differences, and vice versa. A confidence interval to the right of the zero line indicates a positive effect of BRs, while the opposite is a negative effect [27,30]. Forest plots were drawn using GraphPad Prism 9.5.1 software.

## 3. Results

### 3.1. Data Overview

**Figure 2:**
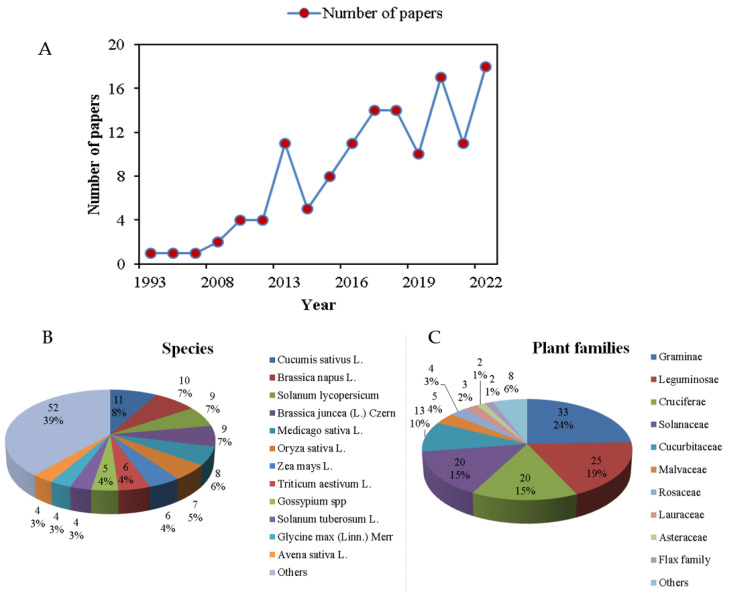
Number of studies, species classification, and family classification. (**A**) The number of papers published each year during the period 1993–2022; (**B**) The percentage of all studied plants; (**C**) The rate of all studied plants in the different families.

This study collected 132 studies regulating plant salt tolerance by exogenous BRs during the period 1993–2022, and the number of research papers in this field climbed rapidly from 2013 (Figure 2A). A total of 48 species were included, among which the most studied plants were *Cucumis sativus* L., *Brassica napus* L., *Solanum lycopersicum*, and *Brassica juncea* (L.) Czern, accounting for 8%, 7%, 7%, and 7%, respectively (Figure 2B, Table S2 (Supplementary File S2)). They were distributed among 18 families, with Graminae accounting for the largest share of 24%, followed by Leguminosae, Solanaceae, and Cruciferae with 19%, 15%, and 15%, respectively (Figure 2C, Table S3 (Supplementary File S2)). A total of 4740 independent studies were obtained for follow-up analysis (Table S1).

### 3.2. Effect of Exogenous BRs on Germination Rate under Salt Stress and Subgroup Analysis

**Figure 3:**
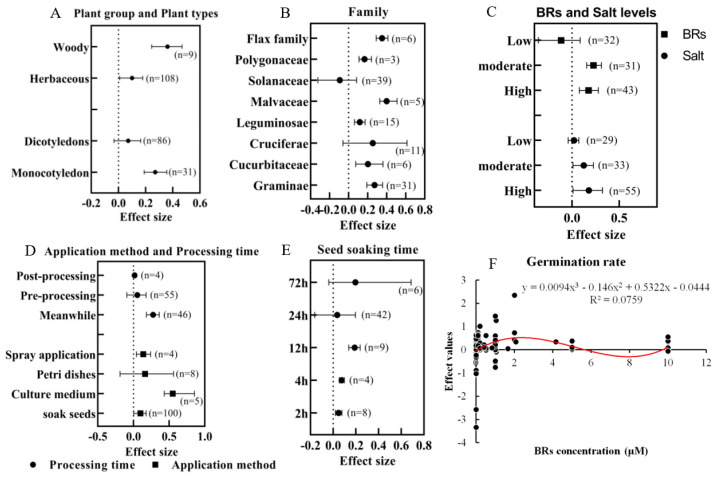
Subgroup analysis of the effect of exogenous BRs on the seed germination rate of plants under salt stress, forest plots are combined effect values (lnR) ± 95% CI, *n* represents the number of independent studies. (**A**) Plant group and plant types, (**B**) family, (**C**) BRs, and salt levels, (**D**) The application method and processing time. Spray application: seeds were treated with BRs by spraying. Petri dishes: BR solution is added to Petri dishes lined with filter paper on which the seeds are laid. Culture medium: BRs are added to the culture medium (a gel containing nutrients) used for seed germination. Soak seeds: seeds are soaked using a solution of BRs. (**E**) Seed soaking time. Confidence intervals do not overlap with the dashed line, indicating a significant difference between the treatment and control groups. (**F**) The effect of BR concentration on the effect value of the germination rate in the 0–10 μM concentration range.

Exogenous BRs increased seed germination under salt stress (Figure 3A), and the subgroup analysis showed that exogenous BRs significantly increased seed germination in both woody and herbaceous plants under salt stress conditions, and the BRs promoted the germination of woody plants significantly more than herbaceous plants. BRs significantly increased the germination rate of monocotyledons (*n* = 31), and the promotion effect was significantly higher than that of dicotyledons (Figure 3A). Exogenous BRs showed a significant promotion of the germination rate in Graminae, Cucurbitaceae, Leguminosae, Malvaceae, Polygonaceae, and Flax family plants. However, there was no significant inhibitory effect on Solanaceae (Figure 3B). Overall, the germination rate showed a trend of increasing and then decreasing with the increase in seed soaking time. The germination rate was significantly higher for 12 h of soaking than for 2 h and 4 h. When the soaking time exceeded 24 h, the BRs did not significantly increase the germination rate of plant seeds under salt stress (Figure 3E). The germination rate was significantly higher and maximum under the same time treatment of salt stress and BRs. While under pretreatment and post-treatment, although BRs played a positive role in the germination rate, only post-treatment had a significant effect.

A categorical analysis of the four application methods revealed that adding BRs to the culture medium, seed immersion, and spraying significantly increased the germination rate under salt stress, with the most significant combined effect value for adding the culture medium, followed by spraying and seed soaking. In contrast, the Petri dish method did not change significantly (Figure 3D). The promotion of BRs was significant (*n* = 33, 55) under medium-level salt (100~150 mM) and high-level salt (NaCl > 150 mM) stresses and the application of BRs was not promoted under low-level salt (NaCl < 100 mM) stress (Figure 3C). The results of this study showed that the germination rate under salt stress varied with the applied concentration of BRs. The low concentration of BRs (<0.02 μM) had no significant effect on seed germination, while medium (0.02~1 μM) and high (>1 μM) concentrations of BRs significantly promoted seed germination. In addition, the promotion of seed germination rate by BRs at the mid-level concentration was significantly higher than that of the low-level (Figure 3C). Regression analysis showed that when the concentration of BRs was in the range of 0~2 μM, the germination rate gradually increased with the increase in BR concentration, and reached the maximum value at two μM, and when BRs > 2 μM, the germination rate firstly decreased and then increased, but did not reach the maximum value (Figure 3F).

### 3.3. Effect of Exogenous BRs on Plant Growth and Biomass under Salt Stress

**Figure 4:**
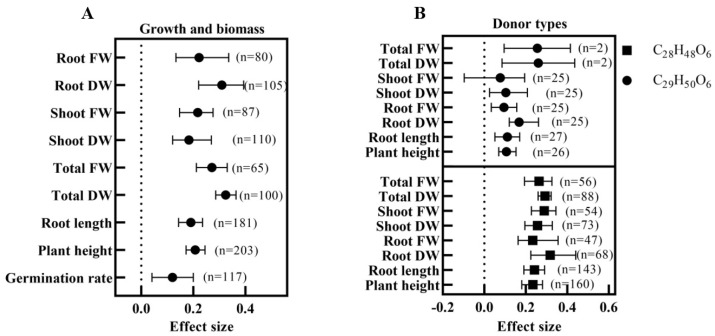
The effect of exogenous BRs on plant growth and biomass under salt stress. (**A**) The effect of BRs on plant height, root length, total dry weight (DW), total fresh weight (FW), shoot DW, shoot FW, root DW, and root FW under salt stress; (**B**) The effect of different donor types of BRs on growth and biomass under salt stress.

Figure 4A shows that exogenous BRs positively affect plant salt tolerance. Under salt stress, the exogenous application of BRs significantly increased plant height, root length, dry weight, and fresh weight. Total DW (E = 0.3244, *n* = 100) had the largest effect value, while shoot DW (E = 0.1827, *n* = 110) had the smallest effect value. Interestingly, the effect values for dry weight were greater than the fresh weight, except for aboveground biomass.

### 3.4. Optimal Application Program for BRs Based on Growth and Biomass Analysis

The extent of the increased plant salt tolerance level by BRs varied according to donor type. Figure 4B shows that C_29_H_50_O_6_ and C_28_H_48_O_6_ significantly increased the plant height, root length, and biomass under salt stress, except for shoot FW. However, the combined effect values of all parameters were higher under the C_28_H_48_O_6_ treatment than C_29_H_50_O_6_, with significant differences in plant height, root length, root FW, and shoot FW.

**Figure 5:**
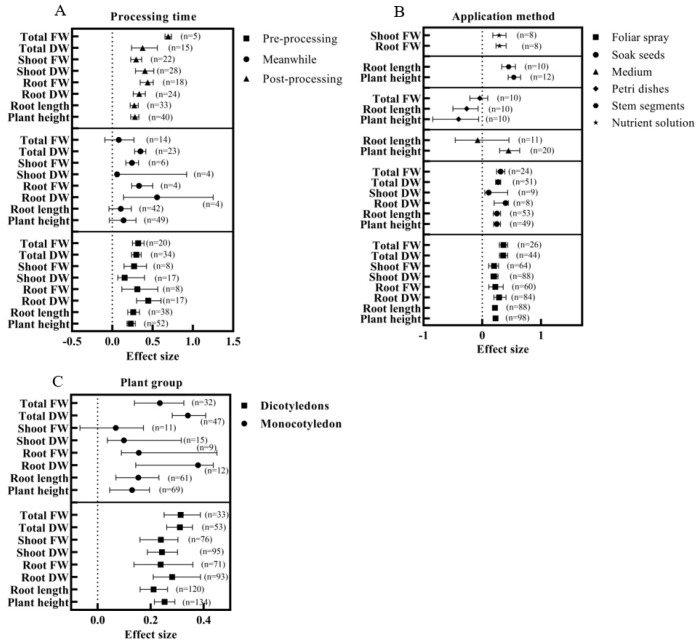
Subgroup analysis of the effect of BRs on plant growth and biomass under salt stress. (**A**) Subgroup analysis for different application periods. (**B**) Subgroup analysis for various application methods, and parameters with some studies of less than eight were removed to ensure the study’s accuracy. (**C**) Subgroup analysis of monocotyledons and dicotyledons.

The effect of BRs on the effect values of growth parameters was related to the period of BR application. Pre-treatment and post-treatment significantly increased the effect values of all growth parameters. In contrast, when BRs were treated simultaneously with NaCl, only the effect values of the total DW, aboveground, and below-ground biomass significantly increased. The combined effect values of plant height, root length, root FW, shoot DW, shoot FW, total DW, and total FW were higher under BRs post-treatment than pretreatment, where the total FW was significantly different. However, the number of studies N was only 5. In addition, the combined effect value of the total FW was significantly higher than the other parameters under post-treatment (Figure 5A).

The effect values of each parameter differed depending on the method of application of BRs (Figure 5B). The effect values of all parameters were significantly higher than the control under foliar spray, seed soaking, stem segment, and nutrient solution usage. Applying BRs in the Petri dish method significantly reduced the plant height and root length under salt stress without significantly affecting the total FW. BRs applied in the medium method significantly increased the plant height under salt stress while not significantly affecting the root length. For shoot FW and root FW, the effect values of nutrient solution usage were higher than those of foliar spray usage, but the differences were insignificant. Regarding plant height and root length, the effect values of stem segment usage were significantly higher than those of seed soaking and foliar spraying. Plant height, root length, and root DW were higher with seed soaking than with foliar spraying, none of which was significant. The shoot DW, total DW, and total FW of foliar sprays were higher than those of soaked seeds, but none were significant.

Exogenous BRs under salt stress enhanced the plant height, root length, and biomass parameters in dicotyledonous and monocotyledonous plants, with significant differences in all parameters in dicotyledonous plants. BRs had no significant effect on the shoot FW of monocotyledons under salt stress (Figure 5C). After applying the BRs under salt stress, the effect values of the root DW and total DW of dicotyledonous plants were lower than those of monocotyledonous plants. At the same time, all other parameters were higher than those of monocotyledonous plants, and the effect values of the plant height of dicotyledonous plants were significantly higher than those of monocotyledonous plants. Overall, the effect values were higher for dicotyledons than for monocotyledons, and the confidence interval ranges were smaller.

**Figure 6:**
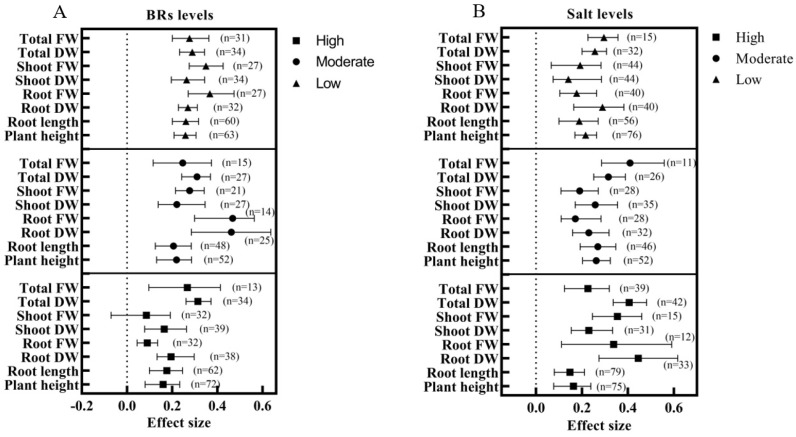
Subgroup analysis of BRs and salt levels based on growth and biomass parameters. (**A**) Subgroup analysis of 3 BR levels, low (BRs < 0.02 μM), medium (0.02–1 μM), and high (BRs > 1 μM); (**B**) Subgroup analysis of 3 salt levels, low (NaCl < 100 mM), medium (100–150 mM) and high (NaCl > 150 mM).

All three concentration levels of BRs significantly increased the plant height, root length, and biomass except for shoot FW (Figure 6A). Overall, the five parameters of plant height, root length, shoot DW, shoot FW, and total FW were higher than the other levels at low levels; the two parameters of root DW and root FW were higher than the other levels at medium levels, and only one parameter of total DW was higher than other levels at high levels. In addition, the maximum effect value of the root FW for the medium-level BRs was 0.468 (*n* = 14). BRs significantly enhanced the plant height, root length, and biomass parameters at all three salt levels (Figure 6B). The effect values of root DW, root FW, shoot FW, and total DW were higher at high levels than at other levels, and the effect values of plant height, root length, shoot DW, and total FW were higher at medium levels than at other levels. Interestingly, the effect values for the parameters at low salt levels did not reach the highest values at all three salt levels. In addition, the effect value of root DW reached a maximum of 0.4449 (*n* = 33) when the BRs were applied under high salt level stress.

### 3.5. Effect of Exogenous BRs on Photosynthesis of Plants under Salt Stress

**Figure 7:**
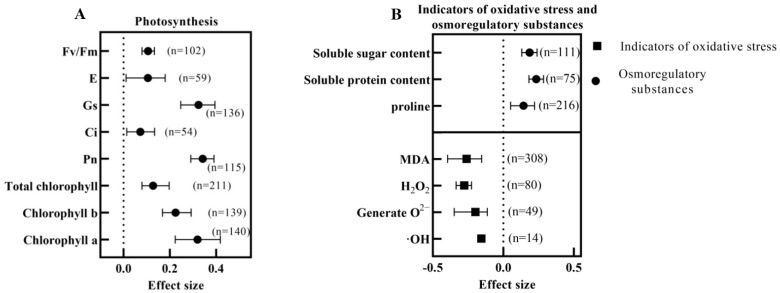
Effect of exogenous BRs on photosynthesis, cell damage, and osmoregulatory substances in plants under salt stress. (**A**) Photosynthesis; maximal photochemical efficiency (Fv/Fm); transpiration rate (E); stomatal conductance (Gs); intercellular CO_2_ concentration (Ci); net photosynthetic rate (Pn); (**B**) Osmoregulatory substance with indicators of oxidative stress; malondialdehyde (MDA).

Applying BRs under salt stress significantly increased the plants’ total chlorophyll, chlorophyll a, chlorophyll b content, Pn, Ci, Gs, E, and Fv/Fm (Figure 7A). The most significant effect value was found for Pn (0.3426, *n* = 115) and the smallest for Ci (0.0731, *n* = 54), and both Pn and Gs had significantly higher effect values than Ci and E. Chlorophyll a > chlorophyll b > total chlorophyll, where BRs promoted chlorophyll a significantly more than the total chlorophyll under salt stress, suggesting that BRs may have a more vigorous regulatory ability on chlorophyll a.

### 3.6. Effect of Exogenous BRs on Oxidative Damage System and Osmoregulatory Capacity of Plants under Salt Stress

**Figure 8:**
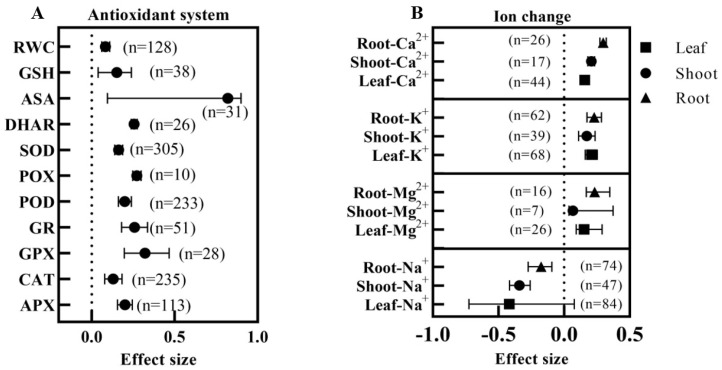
Effect of exogenous BRs on plants’ antioxidant system and cation content under salt stress. (**A**) The antioxidant system, RWC: relative water content, APX: ascorbate peroxidase activity, CAT: catalase activity, GPX: glutathione peroxidase activity, GR: glutathione reductase activity, POD: peroxidase activity, POX: guaiacol peroxidase activity, SOD: superoxide dismutase activity, DHAR: dehydroascorbate reductase activity, ASA: ascorbic acid content, GSH: reduced glutathione content; (**B**) effect of BRs on Na^+^, Mg^2+^, K^+^, and Ca^2+^ content.

Salt stress causes plant cell damage, and BRs can alleviate the damage caused by salt stress. All exogenous applications of BRs significantly reduced the malondialdehyde (MDA), H_2_O_2_, O^2−^·, and ·OH contents of plants under salt stress, with the effect values of H_2_O_2_ content being significantly lower than those of ·OH (Figure 7B). Applying BRs under salt stress significantly increased the enzymatic activities of APX, CAT, GPX (glutathione peroxidase), GR, POD, POX, SOD, and DHAR, and increased the ASA and GSH contents. ASA’s most considerable combined effect value was 0.8206 (*n* = 31). However, the range of its confidence interval was so great that it was not significantly different from the other parameters (Figure 8A). Regarding the enzyme activity, GPX had the most considerable effect value of 0.3215 (*n* = 28), which was significantly higher than CAT and SOD. In addition, BRs also significantly increased the soluble sugar, soluble protein, proline, and relative water content of plants under salt stress (Figure 7B and Figure 8A), where there were no significant differences among osmoregulatory substances in response to BRs.

### 3.7. Effect of Exogenous BRs on the Cation Content of Plants under Salt Stress

As shown in Figure 8B, applying BRs under salt stress significantly increased the Mg^2+^, K^+^, and Ca^2+^ contents and decreased the Na^+^ content in each part (the Leaf—Na^+^ difference was insignificant). There were no significant differences between the different parts of K^+^ and Mg^2+^, while the Ca^2+^ effect values in roots were significantly higher than in the aboveground parts and leaves. Regarding the degree of reduction in plant Na^+^ content by BRs, the most significant effect was observed in leaves, followed by aboveground, and the least in roots. Also, the maximum effect value of Ca^2+^ in the roots was 0.2977 (*n* = 26).

## 4. Discussion

Salt stress, one of the most important abiotic stresses, severely restricts plants’ or crops’ development and yield [6]. Therefore, exploring ways to improve salt tolerance in plants and mechanisms to enhance it is one of the research focuses in this field. Studies have shown that BRs, as the sixth major phytohormone, can significantly improve the salt tolerance of plants [31]. However, there are differences in the conclusions, application methods, doses, and times of BRs in a large number of studies, and a large number of studies have focused on single experiments, which do not provide a global understanding of the effects of BRs on plant seed germination, growth, and physiological biochemistry under salt stress [32,33]. This study will provide a more precise understanding of the optimal usage of BRs and the physiological mechanisms of the effect of BRs on plant salt tolerance on a global scale through Meta-analysis.

### 4.1. Effect of Exogenous BRs on the Germination Rate of Different Plants and the Optimal Method of the Germination Period 

The germination period is the most sensitive to salt stress, and studies have shown that salt stress severely inhibits seed germination [34]. Therefore, increasing the seed germination rate under salt stress is the basis for improving the salt resistance of plants. This study showed that exogenous BRs significantly increased the seed germination rate of plants under salt stress. Further subgroup analysis revealed that exogenous BRs promoted seed germination rates significantly higher in woody plants than herbaceous plants and monocotyledons than dicotyledons. BRs had the greatest promoting effect on seed germination in the flax family and Malvaceae, while no significant inhibitory effect was observed on seed germination in Solanaceae. This indicates that different plants respond differently to BRs, which are more beneficially applied to woody plants, monocotyledons, the Flax family, and Malvaceae, and are not recommended for Solanaceae plants. The reason is presumed to be related to the plant’s characteristics, but the precise mechanism still needs further exploration.

As of the current study, the primary uses of BRs in seeds are spraying [35], Petri dishes [36], culture medium [37], and seed soaking [38]. This study showed the highest germination rate using a culture medium. However, most researchers did not use this method, and only five independent studies used it. Instead, seed soaking (*n* = 100) was the most commonly used method. Therefore, we further analyzed the soaking time and found that the BRs played a tremendous role when the soaking time was 12 h. In summary, seed soaking may be the most convenient to use, but it does not exert the highest effectiveness; therefore, the use of culture medium for BRs should continue to be explored in the future. In this study, we analyzed the effect of BRs on the germination rate of seeds under salt stress under different application periods. We found that the highest germination rate was achieved when BRs were applied simultaneously with NaCl, while the pretreatment of seeds with BRs did not significantly enhance germination under salt stress. BRs applied at concentrations greater than 0.02 μM positively affected the germination rate, and further regression analysis revealed the best effect when BRs were used at 2 μM. Interestingly, the study of salt levels revealed that the higher the salinity level, the more vital it is that BRs contribute to the germination rate. When the NaCl concentration was lower than 100 mM, BRs did not have a significant effect on the increase in germination rate, which might be because the seeds themselves have a certain resistance to salt stress.

### 4.2. Effects of Exogenous BRs on Growth and Biomass of Seedling Plants under Salt Stress and Their Optimal Usage

Plant height, root length, and biomass are the most accurate and straightforward indicators of plant growth, and salt stress can lead to impaired plant growth, mainly in terms of plant height, root length, dry weight, and fresh weight [39,40,41]. Studies show that the application of BRs under salt stress promotes the seedling plant growth and increases biomass [15,42,43]. However, do BRs affect different types of plants differently? What are the primary growth parameters promoted by BRs? What is the optimal type of donor? These questions have yet to be answered. This study showed that all exogenous applications of BRs under salt stress significantly increased plant height, root length, and biomass. The best donor of BRs was C_28_H_48_O_6_, indicating that the activity of C_28_H_48_O_6_ was higher than that of C_29_H_50_O_6_ in exogenously applied plants, which is consistent with the findings of Wani et al. in salt stress on mustard [44]. There was no significant difference in the increases in plant height, root length, and total FW when the BR application was performed at the same time as the salt stress treatment, and the promotion of plant height, root length, root FW, shoot DW, shoot FW, total DW, and total FW by BR treatment after salt stress was higher than in the pretreatment, with the significant difference in total FW indicating that BRs should be applied after salt stress. The main uses of BRs in the seedling stage are foliar sprays, seed soaking, Petri dishes, medium, stem application, and addition to the nutrient solution [16,32,45,46,47]. The analysis of different application methods revealed that Petri dishes and medium usage produced inhibitory effects on plants under salt stress and should be discarded in future studies. The promotion effect of stem segment application was significantly more substantial than that of seed soaking and foliar spraying, and the promotion effect of the nutrient solution usage was higher than that of foliar spraying. However, plant height and root length were the only parameters for comparing stem segment applications. The number of studies was less than 15, and the only parameters involved in the comparison of nutrient application were shoot FW and root FW, and the number of studies was less than 10. Therefore, it is preliminarily assumed that the two uses of the stem segment and nutrient solution may have a more substantial effect, and research in this area should be strengthened to expand the means of using BRs.

In contrast, foliar spraying and seed soaking are the most widely applied uses today [20,48,49]. This study showed no significant difference between foliar spraying and seed soaking for the promotion of plants under salt stress, and both uses had significant effects in terms of promoting various growth and biomass parameters, both of which can be used as BR application methods. In addition, the analysis of monocotyledon and dicotyledon subgroups showed that BRs promoted dicotyledons more than monocotyledons, which may be attributed to the fact that dicotyledons have a large leaf surface planar extension area and a thin cuticle, which tends to be deposited on the foliar surface when sprayed, resulting in the easier uptake of BRs by dicotyledonous plants. This study showed that low levels of BRs promoted the five parameters of plant height, root length, shoot DW, shoot FW, and total FW under salt stress more than other levels, which was the best concentration range for application (BRs < 0.02 μM). High levels of BRs did not significantly affect shoot FW, suggesting that the enhancement of salinity tolerance by an excessively high concentration of BRs began to diminish, which may be related to the inhibitory effect of high concentrations. The effectiveness of BRs was weakest at low salt levels compared to different salt levels, which was similar to the findings of previous studies on the germination rate, indicating that BRs were least effective in improving the salt tolerance of plants when the salt stress level was below 100 mM.

### 4.3. Effect of Exogenous BRs on the Physiological and Biochemical Levels of Plants under Salt Stress

**Figure 9:**
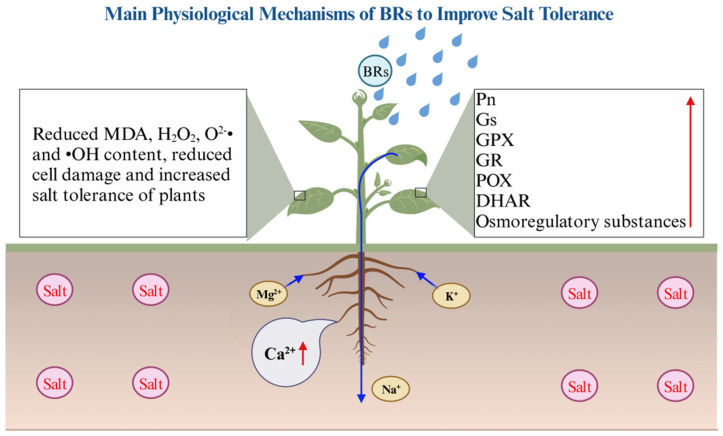
Model of BRs regulating salt tolerance in plants, where red arrows represent promotion. The blue arrows indicate the direction of Na^+^, K^+^, and Mg^2+^ transport. MDA: malondialdehyde, Pn: net photosynthetic rate, Gs: stomatal conductance, GR: glutathione reductase, GPX: glutathione peroxidase, POX: guaiacol peroxidase, DHAR: dehydroascorbate reductase.

It is well known that salt stress increases cellular damage in plants, accelerates superoxide accumulation, and is accompanied by the activation of enzymatic and non-enzymatic systems. At the same time, osmoregulatory substances accumulate, photosynthesis decreases, and ion imbalance occurs, while BRs can promote salt tolerance in plants [50]. There is no way to determine which processes are mainly regulated by BRs, and opposite conclusions exist about the effect of BRs on physiological parameters. The present study showed that the chlorophyll content and photosynthetic fluorescence parameters were significantly increased after the application of BRs under salt stress, with chlorophyll a, Gs, and Pn induced to the greatest extent, indicating that BRs increased photosynthesis mainly by regulating chlorophyll a synthesis, stomatal opening, and the net photosynthetic rate [51]. Compared with salt stress treatment, the exogenous application of BRs induced the synthesis of antioxidant enzymes and antioxidant substances, reduced the production of peroxides and the degree of membrane lipid peroxidation, and alleviated cell damage with ASA, GPX, GR, POX, and DHAR, showing the most significant response to BRs. This indicates that BRs mainly enhance plant salt tolerance by inducing the above substances to scavenge harmful substances [52,53]. However, the confidence interval of ASA is too long, and the error is significant, so further studies on the relationship between BRs and ASA under salt stress are still needed to clarify the physiological functions of BRs. The salt stress can increase plant Na^+^ content and disrupt the ionic balance, producing osmotic stress and ion toxicity, and affecting plants’ normal metabolism [54,55]. In addition, Ca^2+^, as an essential element for plant growth, is involved in regulating plant growth and development, salt stress, and other critical physiological processes [56,57]. The present study showed that applying BRs under salt stress decreased the Na^+^ content and increased the Ca^2+^, K^+^, Mg^2+^, and osmoregulatory substance contents, with the most tremendous increase in Ca^2+^ in roots. This indicates that BRs promote the synthesis of osmoregulatory substances and thus regulate the osmotic balance, reduce the uptake of toxic ions by plants, maintain the ionic balance, and thus promote the improvement in salt tolerance. And, BRs likely promote the production of root Ca^+^ primary signals, which in turn trigger a series of salt tolerance processes (soluble protein, chlorophyll a, stomatal conductance, net photosynthetic rate, and glutathione peroxidase) to regulate plant salt tolerance [58,59]. In addition, BRs can induce root Ca^2+^ signaling, and Ca^2+^ can modulate the interaction between CBL4/CBL8 and CIPK24, which in turn causes changes in the activity of the SOS1 Na^+^/H^+^ antitransporter protein, with a consequent enhancement of the Na^+^ efflux, and ultimately leading to enhanced salt tolerance in the plant [18]. Therefore, CBL4, CBL8, and CIPK24 are essential breakthroughs in breeding salt-tolerant crops. On the other hand, BR signaling is also the focus of salt tolerance in plants, and it was found that the overexpression of *SIBRI1* could enhance BR signaling [60]. In addition, *bHLH*/*HLH* was also related to BR signaling [61]. This suggests that these genes are likely closely related to plant salt tolerance or key genes for breeding salt-tolerant crops. Overall, the salt tolerance regulated by BRs is closely related to Ca^2+^ in the roots and may be the main reason for the increased salt tolerance (Figure 9).

## 5. Conclusions

In this study, 132 papers were screened for meta-analysis to provide evidence that exogenous BRs can improve salt tolerance in plants, and the usage and physiological functions of BRs were further investigated. During the germination period, exogenous BRs significantly increased the germination rate under salt stress, and exogenous BRs promoted the germination rate of woody plants and monocotyledons significantly more than herbaceous plants and dicotyledons, respectively. BRs had the greatest promotion of germination in the flax family and the Malvaceae and had no significant effect on the Solanaceae. The best application method for BRs is the culture medium method, but further research is needed. Seed soaking is commonly used, and its optimal time is 12 h. The best application period for BRs was simultaneously applied with salt stress, and the best concentration was 2 μM. The promotion of salt tolerance by BRs was stronger at high levels of salt concentration than at other levels. At the seedling stage, exogenous BRs significantly increased the plant height, root length, and biomass under salt stress, with the most effect on total DW. The activity of Brassinolide (C_28_H_48_O_6_) was higher than that of Homobrassinolide (C_29_H_50_O_6_), and BR treatment after salt stress was the best application period. The best application method might be stem application, but further study is needed, and there was no significant difference between seed soaking and foliar spraying. The promotion effect of BRs on dicotyledons was higher than that on monocotyledons, and the optimal BR concentration at the seedling stage was recommended to be controlled below 0.02 μM. Exogenous BRs promoted photosynthesis and antioxidant enzyme activity. They increased the accumulation of osmoregulatory substances (soluble protein, soluble sugar, proline) and antioxidant substances (ASA and GSH), thus reducing harmful substances and Na^+^ levels, mitigating cell damage, balancing ion concentrations, and improving plant salt tolerance. BRs regulated soluble proteins, chlorophyll a, Gs, Pn, ASA, GPX, POX, GR, DHAR, and Ca^2+^ in roots most strongly, with BRs regulated Ca^2+^ signals in roots probably being the main reason for improving salt tolerance. BRs first promoted the accumulation of Ca^2+^ in roots, which increased the content of soluble proteins, chlorophyll a, and enzyme activities through the Ca^2+^ signaling pathway, thus improving plant salt tolerance. Finally, we suggest that, in the future, we should also focus on different application methods of BRs, such as culture medium usage at the germination stage, stem segment application at the seedling stage, and nutrient solution usage at the seedling stage. In addition, the mechanism by which BRs promote salt tolerance should be focused on Ca^2+^.

## Data Availability

All data and Supplementary Materials for supporting the findings of this analysis are available from the corresponding author upon the reasonable request.

## Supplementary Materials

The following supporting information can be downloaded at: https://www.mdpi.com/article/10.3390/ijms242216123/s1. References [62,63,64,65,66,67,68,69,70,71,72,73,74,75,76,77,78,79,80,81,82,83,84,85,86,87,88,89,90,91,92,93,94,95,96,97,98,99,100,101,102,103,104,105,106,107,108,109,110,111,112,113,114,115,116,117,118,119,120,121,122,123,124,125,126,127,128,129,130,131,132,133,134,135,136,137,138,139,140,141,142,143,144,145,146,147,148,149,150,151,152,153,154,155,156,157,158,159,160,161,162,163,164,165,166,167,168,169,170,171,172,173] are cited in the Supplementary Materials.
