# Supplementary material for: Promotion of Ca2+ Accumulation in Roots by Exogenous Brassinosteroids as a Key Mechanism for Their Enhancement of Plant Salt Tolerance: A Meta-Analysis and Systematic Review
Source: Int J Mol Sci. 2023 Nov 9;24(22):16123. doi: 10.3390/ijms242216123 (PMC10671333; doi:10.3390/ijms242216123)
Supplement: Supplementary file 1 [file ijms-24-16123-s001.zip › Supplementary File S1.pdf]

Search formulas for different databases：

1. China Science and Technology Journal Database (translate) :

U=(brassinolide OR brassinolide OR 24 epibrassinolide OR 28 homobrassinolide OR 28 epibrassinolide OR brassinosterone OR propionyl brassinolide OR 14 hydroxy brassinolide OR brassinolide OR brassinosteroid) AND U=(salt stress OR salt treatment OR sodium chloride OR NaCl OR salt stress OR sodium salt stress OR sodium salt OR osmotic stress)
Discipline limit: Agricultural Science and Biology

2. CNKI (translate) :

(Subject: brassinolide (accurate)) OR (Subject: brassinolide (accurate)) OR (Subject: 24 epibrassinolide (accurate)) OR (Subject: 28 homobrassinolide (accurate)) OR (Subject: 28 epibrassinolide (accurate)) OR (Subject: brassinosterone (accurate)) OR (Subject: propionyl brassinolide (accurate)) OR (Subject: 14 hydroxy brassinolide sterol (accurate)) OR (Theme: Brassinolide (Precise) OR (Theme: Brassinosterol (Precise)) AND (Theme: Salt Stress (Precise) OR (Theme: Salt Treatment (Precise)) OR (Theme: Sodium Chloride (Precise)) OR (Theme: NaCl (Precise)) OR (Theme: Salt Stress (Precise)) OR (Theme: Sodium Stress (Precise)) OR (Theme: Sodium Salt (Precise)) OR (Theme: Osmotic Stress (Precise))

3. Wanfang Database (translate) :

Subject: (brassinolide OR brassinolide OR 24 epibrassinolide OR 28 homobrassinolide OR 28 epibrassinolide OR brassinosterone OR propionyl brassinolide OR 14 hydroxy brassinolide sterol OR brassinolide OR brassinosterol) AND Subject: (salt stress OR salt treatment OR sodium chloride OR NaCl OR salt stress OR sodium salt stress OR sodium salt OR osmotic stress)

4. Web of Science :

(TS=("Salt Stress" OR "Salt Stresses" OR "Stress, Salt" OR "Salinity Stress" OR "Salinity Stresses" OR "Stress, Salinity" OR "Salinity Stress Reponse" OR "Reponse, Salinity Stress" OR "Salinity Stress Reponses" OR "Stress Reponse, Salinity" OR "Salt Stress Reaction" OR "Reaction, Salt Stress" OR "Salt Stress Reactions" OR "Stress Reaction, Salt" OR "Salt Stress Response" OR "Response, Salt Stress" OR "Salt Stress Responses" OR "Stress Response, Salt" OR "Salinity Stress Reaction" OR "Reaction, Salinity Stress" OR "Salinity Stress Reactions" OR "Stress Reaction, Salinity")) AND TS=("brassinolide" OR "brassinosteroids" OR "brassinosteroid" OR "BR" OR "24-epibrassinolide" OR "28-homobrassinolide" OR "homobrassinolide" OR "EBL" OR "Brassinosterol" OR "Brassinaiide" OR "BRs" OR "24-epicastasterone" OR "BLs" OR "BL" OR "Castasterone" OR "Dolicholide" OR "28-Norbrassinolide" OR "24-Epicastasterone" OR "3a-monofluorohomotyphasterol" OR "5a-MonofluoroHomotyphasterol" OR "5fluoro-28-homocastasterone" OR "28-homocastasterone" OR "Biobras-6" OR "spirostane analogs of BRs" OR "brassinolide, (2alpha,3alpha,5alpha.22R,23R)-isomer" OR "brassinolide, (2alpha,3alpha,5alpha,22S,23S)-isomer" OR "2alpha,3alpha,22alpha,23alpha-tetrahydroxy-24alpha-methyl-B-homo-7-oxa-5alpha-cholestan-6-one")

5. Pubmed :

(("Salt Stress" OR "Salt Stresses" OR "Stress, Salt" OR "Salinity Stress" OR "Salinity Stresses" OR "Stress, Salinity" OR "Salinity Stress Reponse" OR "Reponse, Salinity Stress" OR "Salinity Stress Reponses" OR "Stress Reponse, Salinity" OR "Salt Stress Reaction" OR "Reaction, Salt Stress" OR "Salt Stress Reactions" OR "Stress Reaction, Salt" OR "Salt Stress Response" OR

"Response, Salt Stress" OR "Salt Stress Responses" OR "Stress Response, Salt" OR "Salinity Stress Reaction" OR "Reaction, Salinity Stress" OR "Salinity Stress Reactions" OR "Stress Reaction, Salinity")) AND (("brassinolide" OR "brassinosteroids" OR "brassinosteroid" OR "BR" OR "24-epibrassinolide" OR "28-homobrassinolide" OR "homobrassinolide" OR "EBL" OR "Brassinosterol" OR "Brassinaiide" OR "BRs" OR "24-epicastasterone" OR "BLs" OR "BL" OR "Castasterone" OR "Dolicholide" OR "28-Norbrassinolide" OR "24-Epicastasterone" OR "3a-monofluorohomotyphasterol" OR "5a-MonofluoroHomotyphasterol" OR "5fluoro-28-homocastasterone" OR "28-homocastasterone" OR "Biobras-6" OR "spirostane analogs of BRs" OR "brassinolide, (2alpha,3alpha,5alpha.22R,23R)-isomer" OR "brassinolide, (2alpha,3alpha,5alpha,22S,23S)-isomer" OR "2alpha,3alpha,22alpha,23alpha-tetrahydroxy-24alpha-methyl-B-homo-7-oxa-5alpha-cholestan-6-one"))
