# Supplementary material for: Promotion of Ca2+ Accumulation in Roots by Exogenous Brassinosteroids as a Key Mechanism for Their Enhancement of Plant Salt Tolerance: A Meta-Analysis and Systematic Review
Source: Int J Mol Sci. 2023 Nov 9;24(22):16123. doi: 10.3390/ijms242216123 (PMC10671333; doi:10.3390/ijms242216123)
Supplement: Supplementary file 1 [file ijms-24-16123-s001.zip › Supplementary File S2.pdf]

Table S2 Other species used for Meta-analysis

Others	No. of studies	Others	No. of studies
Vigna unguiculata (Linn.) Walp.	3	Phaseolus vulgaris L.	1
Robinia pseudoacacia L.	3	Nicotiana L	1
Pisum sativum L.	3	Morus alba L.	1
Lolium perenne L.	3	Malus pumila Mill.	1
Hordeum vulgare L.	3	LycopersivonesculentumMill.	1
Cinnamomum camphora (L.) J. Presl	3	Lycium ruthenicum Murray	1
Solanum melongena L.	2	Leymus chinensis (Trin. ex Bunge) Tzvelev	1
Linum usitatissimum L.	2	Lactuca saliva	1
Fragaria ananassa Duch.	2	Festuca elata Keng ex E. B. Alexeev	1
Cucurbita pepo Linn.	2	Fagopyrum esculentum Moench	1
Vitis vinifera L.	1	Eucalyptus urophylla S.T.Blake	1
Vignamungo(L.)Hepper	1	Eriobotrya japonica (Thunb.) Lindl.	1
Triticum turgidum L. var. durum (Desf.) Yan. ex P. C.	1	Dianthus chinensis L.	1
Sorghum bicolor (L.) Moench	1	Cichorium intybus L.	1
Solanum nigrum L.	1	Capsicum frutescens L. (syn. C. annuum L.） var.grossum Bailey.	1
Scutellaria baicalensis Georgi	1	Arachis hypogaea Linn.	1
Populus L.	1	Arabidopsis thaliana (L.) Heynh	1
Pinus elliottii Engelm.	1	Aca-cia decurrens willd var. Mollis Lindl.	1

Table S3 Other families used for Meta-analysis

Others	No. of studies
Caryophyllaceae	1
Labiatae	1
Moraceae	1
Myrtle family	1
Pinaceae	1
Polygonaceae	1
Salicaceae	1
Vitaceae	1
