# Supplementary material for: Promotion of Ca2+ Accumulation in Roots by Exogenous Brassinosteroids as a Key Mechanism for Their Enhancement of Plant Salt Tolerance: A Meta-Analysis and Systematic Review
Source: Int J Mol Sci. 2023 Nov 9;24(22):16123. doi: 10.3390/ijms242216123 (PMC10671333; doi:10.3390/ijms242216123)
Supplement: Supplementary file 1 [file ijms-24-16123-s001.zip › Supplementary File S3.pdf]

Supplementary S3 List of papers used for Meta-analysis data extraction

1. Abd-Allah, E.F.; Alqarawi, A.A.; Hashem, A.; Wirth, S.; Egamberdieva, D. Regulatory roles of 24-epibrassinolide in tolerance of *Acacia gerrardii Benth* to salt stress. *Bioengineered* **2018**, 9, 61-71, doi:10.1080/21655979.2017.1297348.
2. Agami, R.A. Alleviating the adverse effects of NaCl stress in maize seedlings by pretreating seeds with salicylic acid and 24-epibrassinolide. *S. Afr. J. Bot.* **2013**, 88, 171-177, doi:10.1016/j.sajb.2013.07.019.
3. Ahanger, M.A.; Mir, R.A.; Alyemeni, M.N.; Ahmad, P. Combined effects of brassinosteroid and kinetin mitigates salinity stress in tomato through the modulation of antioxidant and osmolyte metabolism. *Plant Physiol. Biochem.* **2020**, 147, 31-42, doi:10.1016/j.plaphy.2019.12.007.
4. Ahmad, H.; Hayat, S.; Ali, M.; Ghani, M.I.; Cheng, Z. Regulation of growth and physiological traits of cucumber (*&ITCucumis sativus&IT* L.) through various levels of 28-homobrassinolide under salt stress conditions. *Can. J. Plant Sci.* **2018**, 98, 132-140, doi:10.1139/cjps-2016-0404.
5. Ahmad, H.; Hayat, S.; Ali, M.; Liu, H.; Chen, X.; Li, J.; Cheng, Z. The Protective Role of 28-Homobrassinolide and *Glomus versiforme* in Cucumber to Withstand Saline Stress. *Plants-Basel* **2020**, 9, doi:10.3390/plants9010042.
6. Ahmad, H.; Hayat, S.; Ali, M.; Liu, T.; Cheng, Z. The combination of *arbuscular mycorrhizal* fungi inoculation (*Glomus versiforme*) and 28-homobrassinolide spraying intervals improves growth by enhancing photosynthesis, nutrient absorption, and antioxidant system in cucumber (*Cucumis Sativus* L.) under salinity. *Ecol. Evol.* **2018**, 8, 5724-5740, doi:10.1002/ece3.4112.
7. Ahmad, P.; Abd Allah, E.F.; Alyemeni, M.N.; Wijaya, L.; Alam, P.; Bhardwaj, R.; Siddique, K.H.M. Exogenous application of calcium to 24-epibrassinosteroid pre-treated tomato seedlings mitigates NaCl toxicity by modifying ascorbate-glutathione cycle and secondary metabolites. *Sci. Rep.* **2018**, 8, doi:10.1038/s41598-018-31917-1.
8. Alam, P.; Albalawi, T.H.; Altalayan, F.H.; Bakht, M.A.; Ahanger, M.A.; Raja, V.; Ashraf, M.; Ahmad, P. 24-Epibrassinolide (EBR) Confers Tolerance against NaCl Stress in Soybean Plants by Up-Regulating Antioxidant System, Ascorbate-Glutathione Cycle, and Glyoxalase System. *Biomolecules* **2019**, 9, doi:10.3390/biom9110640.
9. Ali, B.; Hayat, S.; Fariduddin, Q.; Ahmad, A. 24-Epibrassinolide protects against the stress generated by salinity and nickel in *Brassica juncea*. *Chemosphere* **2008**, 72, 1387-1392, doi:10.1016/j.chemosphere.2008.04.012.
10. Alyemeni, M.N.; Hayat, S.; Wijaya, L.; Anaji, A. Foliar application of 28-homobrassinolide mitigates salinity stress by increasing the efficiency of photosynthesis in *Brassica juncea*. *Acta Bot. Bras.* **2013**, 27, 502-505, doi:10.1590/S0102-33062013000300007.
11. Amraee, L.; Rahmani, F.; Abdollahi Mandoulakani, B. Exogenous application of 24-epibrassinosteroid mitigates NaCl toxicity in flax by modifying free amino acids profile and antioxidant defence system. *Funct. Plant Biol.* **2020**, 47, 565-575, doi:10.1071/FP19191.
12. An, H.; Sheng, W.; Yu, Y.; Zhang, L.; Ceng, H.; Chen, G. Effects of Exogenous 2,4-Epibrassinolide on Physiological Characteristics of Rice Seedlings Under Salt Stress. *Molecular Plant Breeding* **2021**, 19, 2740-2746. doi:10.13271/j.mpb.019.002740.
13. Arora, N.; Bhardwaj, R.; Sharma, P.; Arora, H.K. 28-Homobrassinolide alleviates oxidative

stress in salttreated maize (*Zea mays* L.) plants. *Brazilian Journal of Plant Physiology* **2008**, 20, 153-157, doi:10.1590/S1677-04202008000200007.

14. Azhar, N.; Su, N.; Shabala, L.; Shabala, S. Exogenously Applied 24-Epibrassinolide (EBL) Ameliorates Detrimental Effects of Salinity by Reducing K^+ Efflux via Depolarization-Activated K^+ Channels. *Plant Cell Physiol.* **2017**, 58, 802-810, doi:10.1093/pcp/pcx026.
15. Chang, D.; Yang, Y.; Wang, Y.; Zhang, X.; Zhang, F.; Li, F. Effects of 24-Epi Brassinolide on Seed Germination under Stresses of Salt and PEG in Cotton. *Acta Agriculturae Boreali-occidentalis Sinica* **2015**, 24, 96-101. doi:10.7606/j.issn.1004-1389.2015.03.016.
16. Chen, Y.; Xiang, Y.; Hu, Z.; Gao, Y.; Zhang, Y.; Chen, M.; Khaldun, A.B.M.; Yan, X.; Fan, J. Transcriptomic profiling revealed the role of 24-epibrassinolide in alleviating salt stress damage in tall fescue (*Festuca arundinacea*). *Front. Plant Sci.* **2022**, 13, doi:10.3389/fpls.2022.976341.
17. Chen, Y.; Ge, J.; Liu, Y.; Li, R.; Zhang, R.; Li, K.; Huo, Z.; Xu, K.; Wei, H.; Dai, Q. 24-Epibrassnolide Alleviates the Adverse Effect of Salinity on Rice Grain Yield through Enhanced Antioxidant Enzyme and Improved K^+/Na^+ Homeostasis. *Agronomy-Basel* **2022**, 12, doi:10.3390/agronomy12102499.
18. de Oliveira, V.P.; Roque Lima, M.D.; Serrao Da Silva, B.R.; Batista, B.L.; Da Silva Lobato, A.K. Brassinosteroids Confer Tolerance to Salt Stress in Eucalyptus urophylla Plants Enhancing Homeostasis, Antioxidant Metabolism and Leaf Anatomy. *J. Plant Growth Regul.* **2019**, 38, 557-573, doi:10.1007/s00344-018-9870-3.
19. Ding, H.; Zhu, X.; Zhu, Z.; Yang, S.; Zha, D.; Wu, X. Amelioration of salt-induced oxidative stress in eggplant by application of 24-epibrassinolide. *Biol. Plant.* **2012**, 56, 767-770, doi:10.1007/s10535-012-0108-0.
20. Dong, Y.R.; Zhang, Y.B.; Zhao, D.X.; Geng, B.; Lou, Q.N.; Li, Y.Z.; Wang, Z.H.; Guo, G. Mitigating effect of exogenous 24-epibrassinolide on mulberry seedlings under NaCl stress. *Journal of Nuclear Agricultural Sciences* **2021**, 35, 1466-1475. doi: 10.1016/j.plantsci.2020.110754.
21. Dong, Y.; Wang, W.; Hu, G.; Chen, W.; Zhuge, Y.; Wang, Z.; He, M.R. Role of exogenous 24-epibrassinolide in enhancing the salt tolerance of wheat seedlings. *J. Soil Sci. Plant Nutr.* **2017**, 17, 554-569, doi:10.4067/S0718-95162017000300001.
22. Efimova, M.V.; Khripach, V.A.; Boyko, E.V.; Malofii, M.K.; Kolomeichuk, L.V.; Murgan, O.K.; Vidershpun, A.N.; Mukhamatdinova, E.A.; Kuznetsov, V.V. The Priming of Potato Plants Induced by Brassinosteroids Reduces Oxidative Stress and Increases Salt Tolerance. *Doklady biological Sciences.* **2018**, 478, 33-36, doi:10.1134/S0012496618010106.
23. Efimova, M.V.; Savchuk, A.L.; Hasan, J.A.K.; Litvinovskaya, R.P.; Khripach, V.A.; Kholodova, V.P.; Kuznetsov, V.V. Physiological mechanisms of enhancing salt tolerance of oilseed rape plants with brassinosteroids. *Russ. J. Plant Physiol.* **2014**, 61, 733-743, doi:10.1134/S1021443714060053.
24. Ekinci, M.; Yildirim, E.; Dursun, A.; Turan, M. Mitigation of Salt Stress in Lettuce (*Lactuca sativa* L. var. Crispa) by Seed and Foliar 24-epibrassinolide Treatments. *Hortscience* **2012**, 47, 631-636, doi:10.21273/HORTSCI.47.5.631.
25. El-Mashad, A.A.A.; Mohamed, H.I. Brassinolide alleviates salt stress and increases antioxidant activity of cowpea plants (*Vigna sinensis*). *Protoplasma* **2012**, 249, 625-635, doi:10.1007/s00709-011-0300-7.

26. Fan, C.; Wu, X.; Guan, X.; Zheng, C.; Zhao, H.; Gu, Z.; Liu, W.; Chen, J.; Zheng, Q. Concentration effects and its physiological mechanism of soaking seeds with brassinolide on tomato seed germination under salt stress. *Acta Ecologica Sinica* **2021**, 41, 1857-1867. doi: 10.5846/stxb201905090946.
27. Fariduddin, Q.; Khalil, R.R.A.E.; Mir, B.A.; Yusuf, M.; Ahmad, A. 24-Epibrassinolide regulates photosynthesis, antioxidant enzyme activities and proline content of *Cucumis sativus* under salt and/or copper stress. *Environ. Monit. Assess.* **2013**, 185, 7845-7856, doi:10.1007/s10661-013-3139-x.
28. Fariduddin, Q.; Mir, B.A.; Yusuf, M.; Ahmad, A. 24-epibrassinolide and/or putrescine trigger physiological and biochemical responses for the salt stress mitigation in *Cucumis sativus* L. *Photosynthetica*. **2014**, 52, 464-474, doi:10.1007/s11099-014-0052-7.
29. Fedina, E.O. Effect of 24-epibrassinolide on pea protein tyrosine phosphorylation after salinity action. *Russ. J. Plant Physiol.* **2013**, 60, 351-358, doi:10.1134/S1021443713020088.
30. Furio, R.N.; Salazar, S.M.; Mariotti-Martinez, J.A.; Martinez-Zamora, G.M.; Coll, Y.; Diaz-Ricci, J.C. Brassinosteroid Applications Enhance the Tolerance to Abiotic Stresses, Production and Quality of Strawberry Fruits. *Horticulturae*. **2022**, 8, doi:10.3390/horticulturae8070572.
31. Galal, A. 24-Epibrassinolide Application Enhances Growth and Biochemical Aspects of Squash Under Salt Stress Conditions. *Biologia Futura.* **2018**, 69, 182-196, doi:10.1556/018.69.2018.2.7.
32. Gong, Z.Y.; Hu, Z.H.; Wang, Y.J. Effects of Exogenous EBR on Photosynthetic Physiology of Cowpea Under Waterlogging, Drought and Salt Stress. *Northern Horticulture* **2022**, 9-20. doi:10.11937/bfyy.20221484.
33. Groszyk, J.; Szechynska-Hebda, M. Effects of 24-Epibrassinolide, Bikinin, and Brassinazole on Barley Growth under Salinity Stress Are Genotype- and Dose-Dependent. *Agronomy-Basel* **2021**, 11, doi:10.3390/agronomy11020259.
34. Gupta, P.; Seth, C.S. Interactive role of exogenous 24 Epibrassinolide and endogenous NO in *Brassica juncea* L. under salinity stress: Evidence for NR-dependent NO biosynthesis. *Nitric Oxide-Biol. Chem.* **2020**, 97, 33-47, doi:10.1016/j.niox.2020.01.014.
35. Gupta, P.; Seth, C.S. 24-Epibrassinolide Regulates Functional Components of Nitric Oxide Signalling and Antioxidant Defense Pathways to Alleviate Salinity Stress in *Brassica juncea* L. cv. Varuna. *J. Plant Growth Regul.*, doi:10.1007/s00344-022-10884-y.
36. Gupta, P.; Srivastava, S.; Seth, C.S. 24-Epibrassinolide and Sodium Nitroprusside alleviate the salinity stress in *Brassica juncea* L. cv. Varuna through cross talk among proline, nitrogen metabolism and abscisic acid. *Plant Soil* **2017**, 411, 483-498, doi:10.1007/s11104-016-3043-6.
37. Hayat, S.; Khalique, G.; Wani, A.S.; Alyemeni, M.N.; Ahmad, A. Protection of growth in response to 28-homobrassinolide under the stress of cadmium and salinity in wheat. *Int. J. Biol. Macromol.* **2014**, 64, 130-136, doi:10.1016/j.ijbiomac.2013.11.021.
38. Hayat, S.; Maheshwari, P.; Wani, A.S.; Irfan, M.; Alyemeni, M.N.; Ahmad, A. Comparative effect of 28 homobrassinolide and salicylic of NaCl stress in *Brassica juncea* L. *Plant Physiol. Biochem.* **2012**, 53, 61-68, doi:10.1016/j.plaphy.2012.01.011.
39. He, X.; Wan, Z.; Jin, N.; Jin, L.; Zhang, G.; Lyu, J.; Liu, Z.; Luo, S.; Yu, J. Enhancement of cucumber resistance under salt stress by 2, 4-epibrassinolide lactones. *Front. Plant Sci.* **2022**, 13, doi:10.3389/fpls.2022.1023178.

40. Hegazi, A.M.; El-Shraiy, A.M.; Ghoname, A.A. Mitigation of Salt Stress Negative Effects on Sweet Pepper Using Arbuscular Mycorrhizal Fungi (AMF), *Bacillus Megaterium* and Brassinosteroids (BRs). *Gesunde Pflanz.* **2017**, 69, 91-102, doi:10.1007/s10343-017-0393-9.
41. Hou, H. Effects of Brassinolide on Seed Germination of Rice Under Salt Stress. *Chinese Journal of Tropical Agriculture* **2020**, 40, 1-6.
42. Hu, Y.; Xia, S.; Su, Y.; Wang, H.; Luo, W.; Su, S.; Xiao, L. Brassinolide Increases Potato Root Growth In Vitro in a Dose-Dependent Way and Alleviates Salinity Stress. *Biomed Res. Int.* **2016**, 2016, 8231873. doi:10.1155/2016/8231873.
43. Hua, Z.; Li, X. Effects of Brassinolide on Physiological and Biochemical Characteristics of Scutellaria baicalensis Seedlings under Salt Stress. *Acta Agriculturae Jiangxi* **2021**, 33, 21-26. doi:10.19386/j.cnki.jxnyxb.2021.12.004.
44. Jin-huan, L.; Anjum, S.A.; Mei-ru, L.; Jian-hang, N.; Ran, W.; Ji-xuan, S.; Jun, L.; Xue-feng, Z.; Ashraf, U.; San-gen, W. Modulation of morpho-physiological traits of *leymus chinensis* (trin.) through exogenous application of brassinolide under salt stress. *Journal of Animal and Plant Sciences* **2015**, 25, 1055-1062.
45. Jinlong, L.; Huiling, G.; Lizhou, H.; Changhai, W.; Gengmao, Z.; Xueying, W.; Qingsong, Z. Role of Plant Pigments in the 24-epibrassinolide Ameliorating Salt Stress in *Canola*. *Acta Botanica Boreali-Occidentalia Sinica* **2013**, 33, 90-100, doi:10.3969/j.issn.1000-4025.2013.01.016.
46. Kagale, S.; Divi, U.K.; Krochko, J.E.; Keller, W.A.; Krishna, P. Brassinosteroid confers tolerance in *Arabidopsis thaliana* and *Brassica napus* to a range of abiotic stresses. *Planta* **2007**, 225, 353-364.
47. Karlidag, H.; Yildirim, E.; Turan, M. Role of 24-epibrassinolide in mitigating the adverse effects of salt stress on stomatal conductance, membrane permeability, and leaf water content, ionic composition in salt stressed strawberry (*Fragaria×ananassa*). *Sci. Hortic.* **2011**, 130, 133-140, doi:10.1016/j.scienta.2011.06.025.
48. Kaya, C.; Aydemir, S.; Akram, N.A.; Ashraf, M. Epibrassinolide Application Regulates Some Key Physio-biochemical Attributes As Well As Oxidative Defense System in Maize Plants Grown Under Saline Stress. *J. Plant Growth Regul.* **2018**, 37, 1244-1257, doi:10.1007/s00344-018-9830-y.
49. Khalid, A.; Aftab, F. Effect of exogenous application of 24-epibrassinolide on growth, protein contents, and antioxidant enzyme activities of in vitro-grown *Solanum tuberosum* L. under salt stress. *In Vitro Cell. Dev. Biol.-Plant* **2016**, 52, 81-91, doi:10.1007/s11627-015-9745-2.
50. Kolomeichuk, L.V.; Danilova, E.D.; Khripach, V.A.; Zhabinskyi, V.N.; Kuznetsov, V.V.; Efimova, M.V. Ability of Lactone- and Ketone-Containing Brassinosteroids to Induce Priming in Rapeseed Plants to Salt Stress. *Russ. J. Plant Physiol.* **2021**, 68, 499-509, doi:10.1134/S1021443721020084.
51. Kolomeichuk, L.V.; Khripach, V.A.; Kuznetsov, V.V.; Efimova, M.V. Comparison of Protective Reactions of Rape Seeds to Chloride Salination at Exposure to Epibrassinolide before or during Salt Stress. *Dokl. Biochem. Biophys.* **2022**, 502, 25-29, doi:10.1134/S1607672922010057.
52. Kolomeichuk, L.V.; Efimova, M.V.; Zlobin, I.E.; Kreslavski, V.D.; Murgan, O.K.; Kovtun, I.S.; Khripach, V.A.; Kuznetsov, V.V.; Allakhverdiev, S.I. 24-Epibrassinolide alleviates the toxic effects of NaCl on photosynthetic processes in potato plants. *Photosynth. Res.* **2020**, 146, 151-163, doi:10.1007/s11120-020-00708-z.

53. Kou, J. Effects of 2,4-Epibrassinolide on Germination and Physiological Characteristics of *Avena sativa* L. Seeds under NaCl Stress. *Acta Agrestia Sinica* **2019**, 27, 1562-1568. doi: 10.11733/j.issn.1007-0435.2019.06.013.
54. Kou, J. Effects of Exogenous 2,4-Epibrassinolide on Photosynthetic Characteristics of Oat Seedlings under NaCl Stress. *Acta Agriculturae Boreali-Sinica* **2020**, 35, 79-87. doi:10.7668/hbnxb.20190663.
55. Kou, J. Effects of exogenous 2,4-epibrassinolide on the absorption,transportation and allocation of inorganic ions in *Avena sativa* L.seedlings under NaCl stress. *Chinese Journal of Ecology* **2020**, 39, 855-864. doi:10.13292/j.1000-4890.202003.033.
56. Kou, J. Mitigating effect of exogenous 2, 4-epibrassinolide on the inhibition of oat seed germination under salt stress. *Grassl. Sci.* **2020**, 37, 916-925.
57. Kou, J. Physiological Response of Salt Tolerance of *Medicago sativa* Seedlings Induced by Exogenous 2, 4-Epibrassinolide. *Acta Agriculturae Boreali-Sinica* **2020**, 35, 133-140. doi:10.7668/hbnxb.20191130.
58. Kou, J. Physiological responses of Medicago sativa seed germination induced by exogenous 2,4-epibrassinolide under salt stress. *Grassland and Turf* **2020**, 40, 8-14. doi:10.13817/j.cnki.cyycp.2020.05.002.
59. Kou, J.; Kang, W.; Miao, Y.; Shi, S. Effect of exogenous 2,4-epibrassinolide on the uptake,transport,and disputation of ions,and photosynthetic characteristics of *Medicago sativa* seedlings under NaCl stress. *Acta Prataculturae Sinica* **2016**, 25, 91-103. doi: 10.11686/cyxb2015289.
60. Kou, J.; Kang, W.; Miao, Y.; Shi, S. Effect of exogenous 2,4-epibrassinolide on trace element absorption and chlorophyll fluorescence of *Medicago sativa* L. seedlings under NaCl stress. *Chinese Journal of Eco-Agriculture* **2016**, 0, 345-355. doi:10.13930/j.cnki.cjea.151028.
61. Kou, J.; Shi, S. 2,4-epibrassinolide germination of alfalfa seeds under salt stress and the impact of seedling growth. *Grassland and Turf* **2015**, 35, 1-8. doi: 10.13817/j.cnki.cyycp.2020.05.002.
62. Kou, J.; Shi, S. 2,4-epibrassinolide protection aginest root growth inhibition and oxidative damage of *Medicago sativa* L. seedling under NaCl stress. *Chinese Journal of Eco-Agriculture* **2015**, 23, 1010-1019. doi:10.13930/j.cnki.cjea.150411.
63. Lei, X.; Wan, C.; Tao, J.; Leng, J.; Wu, Y.; Wang, J.; Wang, P.; Yang, Q.; Feng, B.; Gao, J. Effects of soaking seeds with MT and EBR on germination and seedling growth in buckwheat under salt stress. *Acta Agronomica Sinica* **2022**, 48, 1210-1221. doi: 10.3724/SP.J.1006.2022.11040.
64. Li, S.; Zhang, Y.; Yao, Q.; Bai, L.; Hou, L.; Shi, Y. Effects of brassinolide on seedling growth and osmotic regulation characteristics of tomato under iso-osmotic salt stress. *Journal of Northwest A&F University* (*Social Science Edition*) **2020**, 48, 130-136. doi:10.13207/j.cnki.jnwafu.2020.04.017.
65. Li, S.; Zhang, Y.; Yao, Q.; Zhang, Y.; Hou, L.; Shi, Y. Effects of Exogenous BR on Growth and Physiological Resistance of Tomato Seedlings under Different Salt Stresses. *Shandong Agricultural Sciences* **2019**, 51, 50-54. doi:10.14083/j.issn.1001-4942.2019.10.010.
66. Li, T.; Gao, Y.; Ma, X.; Chen, Y.; Wang, Y.; Ma, J. Effects of Exogenous Brassinosteroid on Photosynthesis of Three Spesies of *Populus* under Drought, Salt and Copper Stress. *Genomics and Applied Biology* **2016**, 35, 218-226. doi:10.13417/j.gab.035.000218.
67. Li, W.; Sun, J.; Zhang, X.; Ahmad, N.; Hou, L.; Zhao, C.; Pan, J.; Tian, R.; Wang, X.; Zhao, S.

The Mechanisms Underlying Salt Resistance Mediated by Exogenous Application of 24-Epibrassinolide in Peanut. *International Journal of Molecular Sciences* **2022**, 23, doi:10.3390/ijms23126376.
68. Li, Y.; Sun, S.; Xu, J.; Song, J.; Zhu, L. The alternative oxidase pathway is involved in the BR-induced salt resistance in mustard. *Acta Physiol. Plant.* **2018**, 40, doi:10.1007/s11738-018-2749-x.
69. Liaqat, S.; Umar, S.; Saffeullah, P.; Iqbal, N.; Siddiqi, T.O.; Khan, M.I.R. Protective Effect of 24-Epibrassinolide on Barley Plants Growing Under Combined Stress of Salinity and Potassium Deficiency. *J. Plant Growth Regul.* **2020**, 39, 1543-1558, doi:10.1007/s00344-020-10163-8.
70. Litvinovskaya, R.P.; Shkliarevskyi, M.A.; Kolupaev, Y.E.; Kokorev, A.I.; Khripach, V.A.; Dmitriev, A.P. Effect of 24-Epicastasterone and Its Monosalicylate on Salt Resistance of Arabidopsis thaliana Wild Type and the Salicylate-Deficit NahG Transformants. *Russ. J. Plant Physiol.* **2022**, 69, doi:10.1134/S1021443722020108.
71. Liu, J.; Gao, H.; Wang, X.; Zheng, Q.; Wang, C.; Wang, X.; Wang, Q. Effects of 24-epibrassinolide on plant growth, osmotic regulation and ion homeostasis of salt-stressed canola. *Plant Biol.* **2014**, 16, 440-450, doi:10.1111/plb.12052.
72. Liu, J.; Yang, R.; Jian, N.; Wei, L.; Ye, L.; Wang, R.; Gao, H.; Zheng, Q. Putrescine metabolism modulates the biphasic effects of brassinosteroids on canola and Arabidopsis salt tolerance. *Plant Cell Environ.* **2020**, 43, 1348-1359, doi:10.1111/pce.13757.
73. Lu, X.; Yang, W. Alleviation effects of brassinolide on cucumber seedlings under NaCl stress. *Chinese Journal of Applied Ecology* **2013**, 24, 1409-1414. doi:10.13287/j.1001-9332.2013.0307.
74. Ma, M.; Liu, R.; Zheng, C.; Liu, W.; Yin, X.; Liu, J.; Wang, Z.; Zheng, Q. Regulation of exogenous brassinosteroid on growth of salt-stressed canola seedlings and its physiological mechanism. *Acta Ecologica Sinica* **2015**, 35, 1837-1844. doi: 10.5846/stxb201305231157.
75. Ma, Q.; Gu, W. Regulation of sodium nitroprusside and brassinolide on osmotic adjustment of *Cichorium intybus* L. roots under salt stress. *Jiangsu Agricultural Sciences* **2018**, 46, 99-101. doi:10.15889/j.issn.1002-1302.2018.12.023.
76. Mehmood, S.; Siddiqi, E.H.; Nawaz, I.; Nasir, N. 24-epibrassinolide modulates biomass production, gas exchange characteristics and inorganic nutrients in canola (*Brassica napus* L.) under salt stress. *Pak. J. Bot.* **2022**, 54, 1199-1209, doi:10.30848/pjb2022-4(10).
77. Mokari-Firuzsalari, S.; Khomari, S.; Seyed-Sharifi, R.; Goli-Kalanpa, E.; Azizpour, K. The Combined Influence of Zinc and Epibrassinolide Increase Tolerance to Salt Stress in *Brassica napus* L. *Russ. J. Plant Physiol.* **2019**, 66, 240-249, doi:10.1134/S1021443719020092.
78. Mu, D.; Feng, N.; Zheng, D.; Zhou, H.; Liu, L.; Chen, G. Studies on the Physiological Mechanism of Brassinolide to Improve the Resistance of Rice Seedlings to NaCl Stress. *Water Air Soil Pollut.* **2022**, 233, doi:10.1007/s11270-022-05717-3.
79. Mu, D.; Feng, N.; Zheng, D.; Zhou, H.; Liu, L.; Chen, G.; Mu, B. Physiological mechanism of exogenous brassinolide alleviating salt stress injury in rice seedlings. *Sci. Rep.* **2022**, 12, doi:10.1038/s41598-022-24747-9.
80. Nejad-Alimoradi, F.; Nasibi, F.; Kalantari, K.M. 24-epibrassinolide pre-treatment alleviates the salt-induced deleterious effects in medicinal pumpkin (*Cucurbita pepo*) by enhancement of GABA content and enzymatic antioxidants. *S. Afr. J. Bot.* **2019**, 124, 111-117, doi:10.1016/j.sajb.2019.04.027.

81. Otie, V.; Udo, I.; Shao, Y.; Itam, M.O.; Okamoto, H.; An, P.; Eneji, E.A. Salinity Effects on Morpho-Physiological and Yield Traits of Soybean (*Glycine max* L.) as Mediated by Foliar Spray with Brassinolide. *Plants-Basel* **2021**, 10, doi:10.3390/plants10030541.
82. Pan, L.; Chen, C.; Wang, H.; Zheng, T.; Feng, L.; Jiang, Y. Effect of exogenous substances on seed germination of wild gypsophila under salt stress. *Xiandai Nongcun Keji* **2020**, 77-79.
83. Plazek, A.; Tatrzanska, M.; Maciejewski, M.; Dziurka, M.; Dubert, F. Effects of zearalenone and 24-epibrassinolide on the salt tolerance of selected monocotyledonous crop plants. *J. Appl. Bot. Food Qual.* **2017**, 90, 280-287, doi:10.5073/JABFQ.2017.090.035.
84. Raju, A.D.; Parihar, P.; Singh, R.; Kumar, J.; Prasad, S.M. Synergistic action of indole acetic acid with homobrassinolide in easing the NaCl-induced toxicity in *Solanum melongena* L. seedlings. *Acta Physiol. Plant.* **2020**, 42, doi:10.1007/s11738-020-03054-8.
85. Sadeghi, F.; Shekafandeh, A. Effect of 24-epibrassinolide on salinity-induced changes in loquat (*Eriobotrya japonica* Lindl). *J. Appl. Bot. Food Qual.* **2014**, 87, 182-189, doi:10.5073/JABFQ.2014.087.026.
86. Semida, W.M.; Rady, M.M. Pre-soaking in 24-epibrassinolide or salicylic acid improves seed germination, seedling growth, and anti-oxidant capacity in *Phaseolus vulgaris* L. grown under NaCl stress. *J. Horticult. Sci. Biotechnol.* **2014**, 89, 338-344, doi:10.1080/14620316.2014.11513088.
87. Shahid, M.A.; Pervez, M.A.; Balal, R.M.; Mattson, N.S.; Rashid, A.; Ahmad, R.; Ayyub, C.M.; Abbas, T. Brassinosteroid (24-epibrassinolide) enhances growth and alleviates the deleterious effects induced by salt stress in pea (*Pisum sativum* L.). *Australian Journal of Crop Science* **2011**, 5, 500-510.
88. Shahid, M.A.; Balal, R.M.; Pervez, M.A.; Garcia-Sanchez, F.; Gimeno, V.; Abbas, T.; Mattson, N.S.; Riaz, A. Treatment with 24-epibrassinolide mitigates NaCl-induced toxicity by enhancing carbohydrate metabolism, osmolyte accumulation, and antioxidant activity in *Pisum sativum*. *Turk. J. Bot.* **2014**, 38, 511-525, doi:10.3906/bot-1304-45.
89. Shahzad, R.; Harlina, P.W.; Ewas, M.; Zhenyuan, P.; Nie, X.; Gallego, P.P.; Khan, S.U.; Nishawy, E.; Khan, A.H.; Jia, H. Foliar applied 24-epibrassinolide alleviates salt stress in rice (*Oryza sativa* L.) by suppression of ABA levels and upregulation of secondary metabolites. *J. Plant Interact.* **2021**, 16, 533-549, doi:10.1080/17429145.2021.2002444.
90. Shang, Q.; Song, S.; Zhang, Z.; Guo, S. Exogenous Brassinosteroid Induced the Salt Resistance of Cucumber (*Cucumis sativus* L.) Seedlings. *Scientia Agricultura Sinica* **2006**, 39, 1872-1877.
91. Sharma, I.; Ching, E.; Saini, S.; Bhardwaj, R.; Pati, P.K. Exogenous application of brassinosteroid offers tolerance to salinity by altering stress responses in rice variety *Pusa Basmati-1*. *Plant Physiol. Biochem.* **2013**, 69, 17-26, doi:10.1016/j.plaphy.2013.04.013.
92. Shu, H.M.; Guo, S.Q.; Gong, Y.Y.; Jiang, L.; Zhu, J.W.; Ni, W.C. RNA-seq analysis reveals a key role of brassinolide-regulated pathways in NaCl-stressed cotton. *Biol. Plant.* **2017**, 61, 667-674, doi:10.1007/s10535-017-0736-5.
93. Shu, H.; Guo, S.; Gong, Y.; Ni, W. Effects of brassinolide on leaf physiological characteristics and differential gene expression profiles of NaCl-stressed cotton. *Chinese Journal of Applied Ecology* **2016**, 27, 150-156. doi:10.13287/j.1001-9332.201601.019.
94. Shu, H.; Guo, S.; Gong, Y.; Mamat P.; Ni, W. Effects of Brassinosteroid on Salinity Tolerance of Cotton. *Agricultural Science & Technology* **2014**, 15, 1433-1437. doi:10.16175/j.cnki.1009-4229.2014.09.047.
95. Shu, H.; Guo, S.; Shen, X.; Ni, W. Cotton physiology affected by brassinosteroid under NaCl

stress. *Jiangsu Journal of Agricultural Sciences* **2011**, 27, 1198-1202.
96. Siddiqui, H.; Yusuf, M.; Faraz, A.; Faizan, M.; Sami, F.; Hayat, S. 24-Epibrassinolide supplemented with silicon enhances the photosynthetic efficiency of *Brassica juncea* under salt stress. *S. Afr. J. Bot.* **2018**, 118, 120-128, doi:10.1016/j.sajb.2018.07.009.
97. Singh, S.; Jakhar, S.; Rao, S. Improvement in salt tolerance of *Vigna mungo* (L.) Hepper by exogenously applied 24-epibrassinolide. *Legume Res.* **2020**, 43, 647-652, doi:10.18805/LR-4019.
98. Sivakumar, R.; Priya, S.J. PGRs and nutrient consortium effect on water relations, photosynthesis, catalase enzyme and yield of blackgram under salinity stress. *Legume Res.* **2021**, 44, 413-418, doi:10.18805/LR-4118.
99. Soliman, M.; Elkelish, A.; Souad, T.; Alhaithloul, H.; Farooq, M. Brassinosteroid seed priming with nitrogen supplementation improves salt tolerance in soybean. *Physiol. Mol. Biol. Plants* **2020**, 26, 501-511, doi:10.1007/s12298-020-00765-7.
100. Sousa, D.J.P.; Nogueira, G.A.S.; Teixeira, K.B.S.; Monteiro, G.G.T.N.; Brito, A.E.A.; Nascimento, V.R.; Albuquerque, G.D.P.; Oliveira, T.J.M.; Souza, L.C.; Freitas, J.M.N. et al. Mitigation of the effects of salt stress in cowpea bean through the exogenous aplication of brassinosteroid. *Brazilian journal of biology* **2022**, 82, e260818, doi:10.1590/1519-6984.260818.
101. Sousa, V.Q.; Serra Messias, W.F.; Pereira, Y.C.; Serrao Da Silva, B.R.; Silva Guedes Lobato, E.M.; Alyemeni, M.N.; Ahmad, P.; Da Silva Lobato, A.K. Pretreatment with 24-Epibrassinolide Synergistically Protects Root Structures and Chloroplastic Pigments and Upregulates Antioxidant Enzymes and Biomass in Na^+-Stressed Tomato Plants. *J. Plant Growth Regul.* **2022**, 41, 2869-2885, doi:10.1007/s00344-021-10481-5.
102. Soylemez, S.; Kaya, C.; Dikilitas, S.K. Promotive effects of epibrassinolide on plant growth, fruit yield, antioxidant, and mineral nutrition of saline stressed tomato plants. *Pak. J. Bot.* **2017**, 49, 1655-1661.
103. Su, Q.; Zheng, X.; Tian, Y.; Wang, C. Exogenous Brassinolide Alleviates Salt Stress in Malus hupehensis Rehd. by Regulating the Transcription of NHX-Type $Na^+(K^+)/H^+$ Antiporters. *Front. Plant Sci.* **2020**, 11, doi:10.3389/fpls.2020.00038.
104. Sun, S.; An, M.; Han, L.; Xu, L. Effects of Exogenously Applied 2,4-Epibrassinolide on the Seedlings of *Perennial Ryegrass* under NaCl Stress. *Acta Agrestia Sinica* **2014**, 22, 1045-1050. doi: 10.11733/j.issn.1007-0435.2014.05.020.
105. Sun, S.; An, M.; Han, L.; Yin, S. Foliar Application of 24-Epibrassinolide Improved Salt Stress Tolerance of Perennial Ryegrass. *Hortscience* **2015**, 50, 1518-1523, doi:10.21273/HORTSCI.50.10.1518.
106. Tofighi, C.; Khavari-Nejad, R.A.; Najafi, F.; Razavi, K.; Rejali, F. Physiological and molecular responses of wheat plants to mycorrhizal and epibrassinolide interactions under salinity. *Plant Biosyst.* **2021**, 155, 1075-1080, doi:10.1080/11263504.2020.1829727.
107. Tofighi, C.; Khavari-Nejad, R.A.; Najafi, F.; Razavi, K.; Rejali, F. Responses of wheat plants to interactions of 24-epibrassinolide and *Glomus mosseae* in saline condition. *Physiol. Mol. Biol. Plants* **2017**, 23, 557-564, doi:10.1007/s12298-017-0439-6.
108. Wang, A.; Wang, Y. Effect of brassinolide on the stress resistance in Pinus elliotth engelm seedlings. *Journal of Nanjing Forestry University* (*Natural Sciences*) **1993**, 17, 27-31. doi:10.3969/j.jssn.1000-2006.1993.03.007
109. Wang, D.; Liu, Y.; Zhou, Y.; Li, B.; Nie, S. Physiological regulation of brassinosteroids on seed germination and seedling growth in *Lolium perenne* in response to salt stress. *Pratacultural*

Science **2021**, 38, 1110-1118. doi: 10.11829/j.issn.1001-0629..2021-0034
110. Wang, J.; Zhang, J.; Yue, J.; You, Y.; Zhang, L. BRs, photosynthetic pigments, and chlorophyll fluorescence parameters in *Cinnamomum camphora* seedlings with NaCl stress. *Journal of Zhejiang A & F University* **2017**, 34, 20-27. doi:10.11833/j.issn.2095-0756.2017.01.004
111. Wang, S.; Wang, J.; Zhang, J.; Yue, J. Effects of exogenous 2,4-epibrassinolide on antioxidant enzyme activities of camphor seedlings under salt stress. *Journal of Zhejiang University* **2017**, 43, 476-482. doi: 10.3785/j.issn.1008-9209.2016.09.101.
112. Wang, S.; Zhang, J.; Zhang, L. Effects of exogenous 24-epibrassinolide on chlorophyll content and chlorophyll fluorescence characteristics of camphor seedlings under salt stress. *Journal of Zhejiang University* (*Agriculture & Life Sciences*) **2017**, 43, 45-53.
113. Wang, W.; Ma, D.; Zhao, L.; Ma, Q. Effects of 2,4-table Brassinolide on Enzyme Activity and Root ion Distribution and Absorption in Alfalfa Seedlings. *Acta Agrestia Sinica* **2021**, 29, 1363-1368.
114. Wang, X.; Ji, X.; Liu, L.; Ji, B.; Tian, Y. Effects of epibrassinolide on ion absorption and distribution in Medicago species under NaCl stress. *Acta Prataculturae Sinica* **2018**, 27, 110-119. doi:10.11686/cyxb2017413
115. Wani, A.S.; Hayat, S.; Ahmad, A.; Tahir, I. Efficacy of brassinosteroid analogues in the mitigation of toxic effects of salt stress in *Brassica juncea* plants. *J.Environ.Biol.* **2017**, 38, 27-36, doi:10.22438/jeb/38/1/MS-196.
116. Wei, L.J.; Deng, X.G.; Zhu, T.; Zheng, T.; Li, P.X.; Wu, J.Q.; Zhang, D.W.; Lin, H.H. Ethylene is Involved in Brassinosteroids Induced Alternative Respiratory Pathway in Cucumber (*Cucumis sativus* L.) Seedlings Response to Abiotic Stress. *Front. Plant Sci.* **2015**, 6, 982, doi:10.3389/fpls.2015.00982.
117. Wei, S.; Ji, B.; Li, Z.; Gu, W. Effect of brassinolide on physiological characteristics of maize seedlings under salt stress. *Journal of Northeast Agricultural University* **2018**, 49, 9-16. doi:10.19720/j.cnki.issn.1005-9369.2018.05.002.
118. Wu, X.; Cha, D.; Zhu, Z.; Li, X. Effects of Exogenous 24-Epibrassinolide on Seed Germination, Physiological Characteristics of Eggplant Seedlings under NaCl Stress. *Plant Physiology Journal* **2011**, 47, 607-612. doi:10.13592/j.cnki.ppj.2011.06.006.
119. Yan, H.; Peng, Y.; Zhao, X.; Lu, Y. Effect of Exogenous 24-epibrassinolide on Seed Germination and Seedling Growth of Maize Under Different Stress. *Journal of Nuclear Agricultural Sciences* **2016**, 0, 988-996. doi:10.11869/j.issn.100-8551.2016.05.0988
120. Yang, W.W.; Liu, Y.; Nie, S.M. Effect of Exogenous Brassinosteroids on Germination of Tomato Seeds under Salt Stress. *Horticulture & Seed* **2022**, 42, 43-46, doi:10.16530/j.cnki.cn21-1574/s.2022.11.017.
121. Yuan, H.; Guo, W.; Zhao, L.; Yu, Y.; Cheng, L.; Chen, S.; Kang, Q.; Song, X.; Chen, J.; Wu, G. Effect of exogenous brassinolide treatment on seed germination and seedling physiological characteristics of flax under NaCl stress. *Journal of Northeast Agricultural University* **2019**, 50, 11-16. doi:10.19720/j.cnki.issn.1005-9369.2019.11.0002.
122. Yue, J.; Fu, Z.; Zhang, L.; Zhang, Z.; Zhang, J. The Positive Effect of Different 24-epiBL Pretreatments on Salinity Tolerance in *Robinia pseudoacacia* L. Seedlings. *Forests* **2019**, 10, doi:10.3390/f10010004.
123. Yue, J.; You, Y.; Zhang, L.; Fu, Z.; Wang, J.; Zhang, J.; Guy, R.D. Exogenous 24-Epibrassinolide Alleviates Effects of Salt Stress on Chloroplasts and Photosynthesis in

Robinia pseudoacacia L. Seedlings. *J. Plant Growth Regul.* **2019**, 38, 669-682, doi:10.1007/s00344-018-9881-0.
124. Yue, J.; Zhang, J.; You, Y.; Wang, J.; Zhang, L.; Fu, Z.; Wang, S.; Yi, X. Effects of Brassinostreoids on photosynthesis and ultrastructure of chloroplasts in *Robinia pseudoacacia* seedlings under salt stress. *Journal of Northwest A & F University(Natural Science Edition)* **2017**, 45, 56-66. doi:10.13207/j.cnki.jnwafu.2017.10.008.
125. Yusuf, M.; Fariduddin, Q.; Khan, T.A.; Hayat, S. Epibrassinolide reverses the stress generated by combination of excess aluminum and salt in two wheat cultivars through altered proline metabolism and antioxidants. *S. Afr. J. Bot.* **2017**, 112, 391-398, doi:10.1016/j.sajb.2017.06.034.
126. Zhang, S.; Luo, S.; Zhang, W.; Li, J.; Zhang, G. Effects of exogenous 2, 4- epibrassinolide on growth and photosynthetic physiological characteristics of cucumber seedlings under cadmium stress. *Acta Botanica Boreali-Occidentalia Sinica* **2022**, 42, 272-279. doi:10.19720/j.cnki.issn.1005-9369.2022.06.002.
127. Zhao, X.; Xu, S.; Li, Q.; Ye, P. Effects of EBR Immersion on Seed Germination of *Lycium ruthenicum* under Salt Stress. *For. Sci. Technol.* **2021**, 59-61. doi:10.13456/j.cnki.lykt.2020.03.24.0001.
128. Zheng, C.; Fan, C.; Zheng, Q.; Liu, W.; Chen, J.; Ding, W.; Li, P. Growth of tomato seedlings under salt stress with external application of 2,4-epibrassinolide and the influence of physiological characteristics. *Journal of Zhejiang Agricultural Sciences* **2022**, 63, 991-995. doi:10.16178/j.issn.0528-9017.20212750.
129. Zheng, Q.; Liu, J.; Liu, R.; Wu, H.; Jiang, C.; Wang, C.; Guan, Y. Temporal and spatial distributions of sodium and polyamines regulated by brassinosteroids in enhancing tomato salt resistance. *Plant Soil* **2016**, 400, 147-164, doi:10.1007/s11104-015-2712-1.
130. Zhou, N. Effect of 24-Epibrassinolide on Germination of Cucumber Seeds under Salt Stress. *Journal of Hainan Tropical Ocean University* **2016**, 23, 66-68. doi:10.13307/j.issn.1008-6722.2016.02.14.
131. Zhou, Y.; Luan, X.; Wang, L.; Zhang, Z.; Hui, Z. Effects of EBR Pretreatment on Antioxidant Substances and Enzyme Activities of *Grapevine* Seedling Leaves under Salt Stress. *Acta Botanica Boreali-Occidentalia Sinica* **2018**, 38, 291-297. doi:10.7606/j.issn.1000-4025.2018.02.0291.
132. Zhu, T.; Deng, X.; Tan, W.; Zhou, X.; Luo, S.; Han, X.; Zhang, D.; Lin, H. Nitric oxide is involved in brassinosteroid-induced alternative respiratory pathway in *Nicotiana benthamiana* seedlings' response to salt stress. *Physiol. Plant.* **2016**, 156, 150-163, doi:10.1111/ppl.12392.
